# Root-inspired grafting of wood surfaces with hyperbranched polymers for enhanced interfacial adhesion with impregnated decorative paper†

**DOI:** 10.1039/d4ra07688e

**Published:** 2024-12-09

**Authors:** Hui Li, Ru Liu, Xinyu Li, Kun Xu, Jinzhen Cao

**Affiliations:** a School of Materials Science and Technology, Beijing Key Laboratory of Wooden Material Science and Application, Beijing Forestry University Haidian Beijing 100083 China caoj@bjfu.edu.cnw; b Research Institute of Wood Industry, Chinese Academy of Forestry Haidian Beijing 100091 China liuru@criwi.org.cn; c Guangdong Tianyuan Huibang New Materials Co., Ltd Foshan Guangdong 523000 China

## Abstract

A root-like waterborne hyperbranched polymer, synthesized from diethylenetriamine (DETA) and methyl acrylate (MA) monomers, was inspired by the effect of solidifying soil with tree roots. This polymer was then blended with aqueous isocyanate SK615, known as MD-HBP-NH_2_, to serve as a surface modifier for blockboards. The blockboards were treated with a modifier to enhance the interfacial adhesion with melamine-formaldehyde (MF) resin-impregnated decorative paper, thereby preventing surface cracks. The polycondensation reaction temperatures of the modifiers were compared. These results indicated that a hyperbranched root-structured polymer emulsion was formed through Michael addition reactions. Following this modification, the blockboards demonstrated enhanced planeness and dimensional stability. Furthermore, the isocyanate groups reacted with the exposed hydroxyl groups, and the amino groups reacted with the aldehyde groups in the MF resin, thereby enhancing the interfacial bonding strength between the wood and the impregnated decorative paper. At a polycondensation temperature of 155 °C, optimal overall performance was attained, with the ability to penetrate the wood surface to a depth of 1.28 mm, and exhibited superior surface crack resistance. Moreover, this waterborne hyperbranched polymer modifier is eco-friendly, green, and non-toxic, with lower levels of volatile organic compounds. This presents a promising avenue for the development of eco-friendly modifiers to prevent surface cracking in wood-based panels with impregnated decorative paper.

## Abstract 2

A root-like waterborne hyperbranched polymer, synthesized from diethylenetriamine (DETA) and methyl acrylate (MA) monomers, was inspired by the effect of solidifying soil with tree roots.

## Graphical abstract

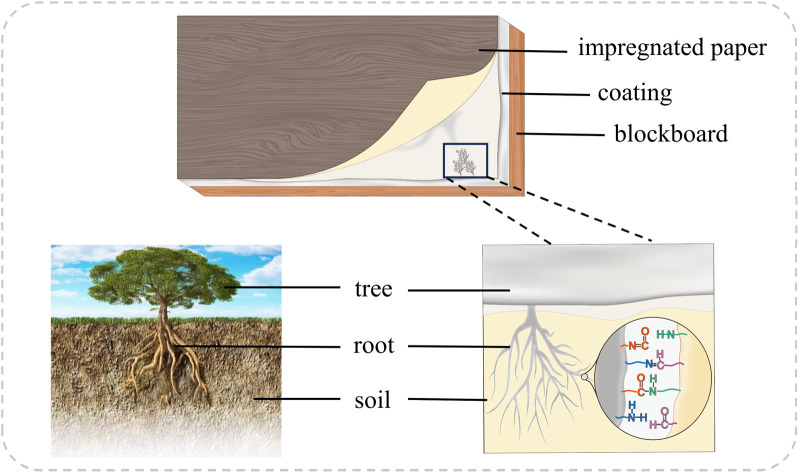

## 1 Introduction

Global climate change has significantly affected the wood industry in recent years. Wood, known as a ‘carbon-negative’ material, offers a climate-friendly solution by storing carbon and substituting fossil products and fuels.^1,2^ In addition to its renewability, the diverse material properties and unique environmental characteristics^3–5^ of wood make it widely used in furniture decoration and renovation. In recent years, wood-based panels have become a predominant choice for decoration and construction. Conversely, traditional wood-based panels exhibit poor surface decoration,^6^ necessitating additional treatment for practical applications. Impregnated decorative paper, typically utilizing melamine-formaldehyde (MF) resin as the impregnating adhesive, is commonly employed. MF resin offers various advantages, such as being colorless, transparent, possessing high adhesive strength, and being wear-resistant and heat-resistant.^7^ Consequently, impregnated decorative paper is experiencing rapid development. As a significant component of wood-based panels, impregnated decorative paper veneer plywood and blockboards have gained consumer favor since 2010 owing to their resemblance to solid wood patterns. They currently hold a market share of 50–70% and exhibit promising application prospects. However, plywood and blockboards often experience surface cracking when bonded with impregnated decorative papers. This issue may result from the poor flatness and dimensional stability of the surface veneer, as well as the weak interfacial bonding between the wood surface and the impregnated decorative paper.

Dimensional instability in wood arises from fluctuating moisture levels,^8^ primarily because of the abundance of hydroxyl groups present in cellulose, hemicellulose, and lignin on the wood surface.^9–11^ The hydroxyl groups serve as potential sites for chemical modification. Polymer graft modifications, such as polyurethane graft modification, can enhance the dimensional stability of wood surfaces by leveraging their high reactivity with hydroxyl groups.^12^ Waterborne polyurethane (WPU) adhesives offer numerous advantages, including low-temperature flexibility, resistance to acids and alkalis, excellent solvent resistance, superior weatherability, and low levels of volatile organic compounds (VOCs).^13–15^ They have been extensively studied and applied in various fields including coatings, furniture, and wood processing.^16^ Chen *et al.*^17^ applied silicone-complexed waterborne polyurethane–polyacrylate to antique wood surface finishing, forming strong hydrogen bonds with the hydroxyl groups of wood, thereby improving dimensional stability, waterproofness, and solvent resistance.

Certain natural structures, such as twigs, snowflakes, leaves, and roots, have evolved through environmental selection, rendering them well-suited to their habitats. Similarly, hyperbranched polymers have gained considerable interest from researchers owing to their intricate three-dimensional structures, abundant terminal functional groups, and straightforward synthesis methods.^18–20^ This highly branched architecture minimizes interactions and entanglements, leading to good solubility, low viscosity, and reduced crystallinity, solvent usage, costs, and emissions when applied to wood surfaces.^21,22^ Therefore, the application of hyperbranched polymers enhances performance by increasing the solid content and reactivity with wood.^23,24^ Several studies have investigated waterborne hyperbranched polymers in wood modification, including their applications in wood-based panel adhesives,^25–27^ surface coatings,^28,29^ and impregnation.^30–32^ These studies typically involved synthesizing aqueous hyperbranched compounds and either utilizing them directly or blending them with other compounds to enhance wood properties and bonding strength with other products. Nevertheless, no reports have explored the surface treatment of wood with hyperbranched polymers to improve the surface quality and enhance the interfacial bond strength with impregnated decorative paper.

To address common surface cracking issues, this study aimed to improve both the dimensional stability of the surface veneer and the interfacial bonding strength between wood and impregnated decorative paper. Inspired by the soil-solidifying effect of tree roots, we synthesized a highly reactive aqueous hyperbranched polyurethane compound (MD-HBP-NH_2_). This compound combines the excellent properties of hyperbranched polymers and polyurethanes. It was grafted onto the wood veneer surface to create a “tree root” like structure, thereby improving surface quality attributes such as flatness and dimensional stability. Throughout the hot-pressing process, the abundant end groups of the hyperbranched polymer infiltrate the impregnated decorative paper, similar to the “soil”. This resulted in a physicochemical anchoring effect that enhanced the interfacial bonding strength between the substrate and paper, thus preventing cracks. This study introduced a novel avenue for developing an environmentally friendly modifier to combat surface cracking in wood-based panels adorned with impregnated decorative paper.

## 2 Materials and methods

### 2.1 Materials

Diethylenetriamine (C_4_H_13_N_3_, reagent grade ≥99%), methylacrylate (C_4_H_6_O_2_, reagent grade ≥99%), and methanol (CH_3_OH, reagent grade ≥99%) were purchased from Shanghai McLean Biology Co. Ltd (Shanghai, China). The water-based crosslinking agent SK-615, with an-NCO content of (6.85 ± 0.35)% and solid content of (50 ± 1)%, was obtained from Yancheng Shengkang New Material Technology Co. (Yancheng, China). The thickening and defoamer agents were purchased from Guangdong Zhongfederal Fine Chemical Co., Ltd and Dongguan Laibao Packaging Technology Co., Ltd, respectively (Dongguan, China). The Chinese fir veneer (*Cunninghamia lanceolata* Hook.) surfaced blockboards measuring 1220 mm × 2440 mm × 18 mm served as the substrate panel and were sourced from Dehua Rabbit Baby Decoration New Materials Co., Ltd (Deqing, China). The melamine-formaldehyde impregnated decorative paper utilized in this study was obtained by impregnating neat decorative papers with a melamine-formaldehyde adhesive (MF) at a concentration of 125 g m^−2^, along with other chemicals such as curing agents, wetting agents, and release agents. The study featured a dark gray wood grain design and was supplied by Guangdong Tianyuan Huibang New Materials Co., Ltd (Foshan, China).

### 2.2 Synthesis of amino-terminated hyperbranched polymer (HBP-NH_2_)

**Fig. 1:**
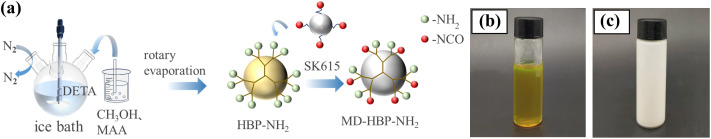
(a) Synthetic pathways for HBP-NH_2_ and MD-HBP-NH_2_. (b) HBP-NH_2_. (c) MD-HBP-NH_2_.

According to the method outlined in previous studies, an amino-terminated hyperbranched polymer (HBP-NH_2_) was synthesized from diethylenetriamine and methylacrylate. As depicted in Fig. 1, 52 mL of diethylenetriamine was placed in a 250 mL three-necked flask, which was then cooled in an ice-water bath. Subsequently, a mixture of 43 mL methyl acrylate and 100 mL methanol was slowly added using a constant-pressure funnel under N_2_ protection. The molar ratio of the reaction feed was controlled to be diethylenetriamine (DETA) : methyl acrylate (MA) = 1.2 : 1. After addition, colorless AB_3_ and AB_2_ monomers were obtained, which were then stirred at 300 rpm for 4 hours reaction at room temperature. The monomers were then transferred to a rotary evaporator circular bottom flask and methanol was removed *via* reduced-pressure distillation. The reaction was continued under vacuum at an elevated temperature until a viscous light-yellow terminal amino hyperbranched compound, HBP-NH_2_, was obtained. The samples were categorized into five groups based on the reaction temperature: 140 °C, 145 °C, 150 °C, 155 °C, and 160 °C.

### 2.3 Synthesis of wood modification agent (MD-HBP-NH_2_)

Initially, 2 g of HBP-NH_2_ and 18 g of the aqueous crosslinking agent SK-615 were added to a beaker. Subsequently, they were dispersed in 46.66 g of deionized water. To control the foam and enhance the viscosity, 0.33 g of a defoamer agent and 0.33 g of a thickening agent were introduced into the system. The reactive group content (–NCO) of aqueous SK-615 was 8.5 ± 0.2%, with a solid content of 50 ± 1%. The reactive group content (polyether-modified silicone oil) of the defoamer was ≥65%. The solid content of the acrylic thickening agents was 80%. Mechanical stirring was then employed to achieve uniformity, resulting in a milky white emulsion. Digital images of HBP-NH_2_ and MD-HBP-NH_2_ are shown in Fig. 1(b) and (c), respectively.

### 2.4 Production of surface decorated blockboard with paper impregnated melamine-formaldehyde resin (I-GB)

**Fig. 2:**
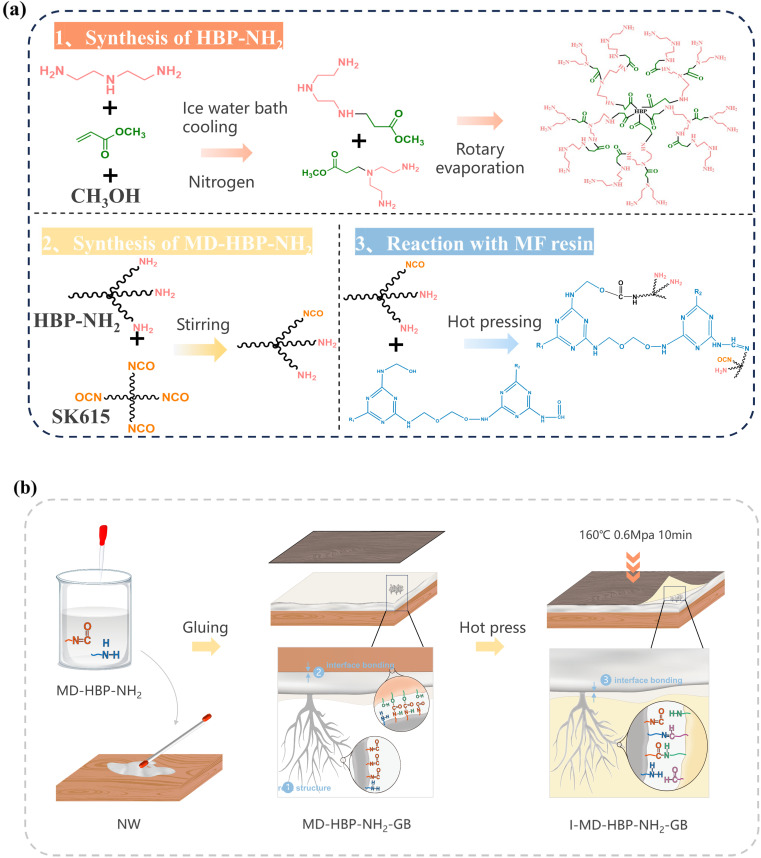
(a) Synthesis and reaction process of MD-HBP-NH_2_. (b) The preparation procedure for a wood/impregnated grafted blockboard sample (I-MD-HBP-NH_2_-GB).

The synthesis of MD-HBP-NH_2_ is illustrated in detail in Fig. 2(a). First, MD-HBP-NH_2_ was grafted onto a blockboard substrate using a coating rod with a wet-coating thickness of 40 μm. The grafted blockboard was cured in an oven at 60 °C for 15 min. Subsequently, the blockboard was surface-coated twice with MD-HBP-NH_2_ to produce the grafted blockboards (GB). Finally, the melamine-formaldehyde impregnated decorative paper was laminated onto the grafted blockboards *via* hot pressing at 160 °C and 0.6 MPa for 10 min. The reaction pathway illustrated in Fig. 2 involves diethylenetriamine (DETA), methyl acrylate (MA), and methanol as starting materials, undergoing a three-step reaction: Michael addition, polycondensation, and isocyanate grafting. Michael addition polymerizations of amines and acrylic monomers are versatile approaches for synthesizing biomaterials with various applications.^33^ This route can offer certain advantages, such as fewer synthetic steps, higher reaction yields, and improved operability. Additionally, the figure depicts the addition polymerization and aldehyde-amine condensation reactions between MD-HBP-NH_2_ and MF resin. The preparation process of I-MD-HBP-NH_2_-GB and a schematic diagram of the interfacial chemical bonding are shown in Fig. 2(b). For comparative analysis, untreated wood subjected to hot pressing (I-NW) and wood treated with SK615 as a coating followed by hot pressing (I-SK615-GB) were used as control groups (I-NW and I-SK615-GB). These controls enabled a direct assessment of the effects of MD-HBP-NH_2_ grafting and the application of decorative paper on the blockboard's properties and performance. Both MD-HBP-NH_2_ and SK615 were applied using a coating rod with a film thickness of 40 μm. To ensure uniform coverage, the coating was applied in two layers.

### 2.5 Chemical structure analysis

Fourier-transform infrared (FTIR, Nicolet iN10-MX, Thermo-Scientific, USA) spectra with a resolution of 4 cm^−1^ were used to analyze the chemical group alterations in the specimens using an attenuated total reflectance (ATR) mode. For FTIR analysis of MF resin, the impregnated paper powder was dried and tested in infrared using the KBr tablet method. The powder was thoroughly dried, mixed with KBr at a mass ratio of 1 : 200, and pressed into tablets. The analyzed samples included monomers, HBP-NH_2_, MD-HBP-NH_2_, MD-HBP-NH_2_ with melamine-formaldehyde impregnated decorative paper, and melamine-formaldehyde impregnated decorative paper. ^1^H NMR spectra were acquired using an AVANCE III 400 spectrometer (Bruker, Germany) to detect functional group changes in HBP-NH_2_ and MD-HBP-NH_2_. For liquid ^1^H NMR, HBP-NH_2_ and MD-HBP-NH_2_ were dissolved in deuterated chloroform. For solid ^1^H NMR analysis of MF resin, the impregnated paper was ground into powder and dried, and then a small amount of the dried sample was placed in an NMR tube. Deuterated chloroform was added, and the sample was ultrasonicated to dissolve the powder before conducting ^1^H NMR testing. Additionally, XPS analyses were conducted on unmodified and MD-HBP-NH_2_ modified wood specimens using a K-Alpha instrument (Thermo Scientific, USA) to determine the number of functional groups present on the wood surface.

### 2.6 Scanning electron microscopy (SEM)

The impregnated decorative paper-decorated and grafted blockboards were oven dried at 60 °C for 24 h. Scanning electron microscopy (SEM, Regulus8100, Hitachi Science System, Japan) was employed to examine the micromorphology of the coatings, surfaces for bonding strength tests, and end face of the I-GB. Additionally, energy-dispersive spectroscopy (EDS), attached to the SEM, was utilized to investigate the infiltration behavior of nitrogen in specimens representing MD-HBP-NH_2_ of I-GB.

### 2.7 Transmission electron microscope (TEM)

A suspension of the MD-HBP-NH_2_ specimen was prepared in distilled water. A drop of the diluted suspension was placed on a carbon-coated copper grid and dried in an oven at 60 °C. Particle morphology was observed using a transmission electron microscope (TEM, HT7800, Hitachi, Japan) operating at 120 kV.

### 2.8 Particle size and molecular weight

The particle size and molecular weight of HBP-NH_2_ were measured at room temperature. Particle size was determined using a laser particle size analyzer (Zetasizer Nano ZS, Malvern, UK). Before measurement, samples from each group were diluted with deionized water to a mass fraction of 0.005% and ultrasonically dispersed for 10 min. The dispersion was then injected into the Malvern potentiostat cell and analyzed using the laser particle size analyzer. The molecular weight of HBP-NH_2_ was determined by gel permeation chromatography (GPC) using a Waters GPC 1515 system (USA). The polymers were dissolved in DMSO prior to analysis. The number average molecular weight (*M*_n_) of HBP-NH_2_ was measured using a Shimadzu RID-20 GPC system equipped with a RID-20 oscillometric refractive detector (Shimadzu Co., Inc., Japan) and a TSKgel GMPWxl column (molecular weight range: 300–1 000 000). Measurements were conducted at 35 °C, using a narrow-distribution polyethylene glycol (PEO) standard (molecular weights: 600–903 000) for calibration. The mobile phase consisted of 0.1 M NaN_3_ + 0.06% NaN_3_ aqueous solution at a flow rate of 0.6 mL min^−1^.

### 2.9 Surface properties

The surface cracking resistance and surface cold and hot cycling resistance of the I-GB were evaluated based on the standard “Test methods of evaluating the properties of wood-based panels and surface decorated wood-based panels” (GB/T 17657-2022). For the surface cracking resistance test, I-GB specimens measuring 200 × 180 mm were heated in an oven at 70 °C for 24 h and then stored at 23 °C and 50% RH until equilibrium was reached. In the surface cold and hot cycling resistance tests, I-GB specimens measuring 100 × 100 mm were heated in an oven at 80 °C for 2 h and immediately transferred to a low-temperature test chamber at −20 °C for 2 h, constituting one cycle. After four cycles, the specimens were left at room temperature for over 1 h. The surface changes were then observed using a 6× magnifying glass under illuminance of 800–1000 l× and recorded. Three replicates were tested for each group.

The surface bonding strength of the impregnated decorative paper-decorated blockboards was measured using a multifunctional mechanical testing machine (AG-2000A, Shimadzu, Japan). Initially, a hot-melting adhesive was evenly applied to the bottom surface of the metal head grip and bonded to the center of the test specimens, which were I-GBs with dimensions of 50 mm × 50 mm. When the hot-melt adhesive was cooled to room temperature, the metal head grip along with the specimen was installed in the fixture and loaded perpendicular to the bonding surface. The specimens were then destroyed within 60 s at a loading speed of 1 mm s^−1^ during testing. Subsequently, the maximum failure load value was calculated, and the results were averaged for the six different specimens. Photographs of the cracks were captured at 4× magnification for observation using an optical microscope (S6D; Leica Microsystems Co., Ltd, Germany).

### 2.10 Physical performance tests

Following the standard “Test Methods for Physical and Chemical Properties of Wood-based Panels and Decorative Wood-based Panels” (GB/T 17657-2022), the dimensional stability of GB was evaluated as follows. The specimens from each group of GB were divided into two sets for dry heat and high humidity tests. The transverse and longitudinal lengths of each specimen were measured before and after the tests, respectively.

For the dry heat test, six test specimens were oven-dried at 70 °C for 24 h. Subsequently, the specimens were immediately transferred to a desiccator for 1 h to prevent moisture.

For high-humidity testing, six additional specimens were placed in a constant temperature and humidity box set to 40 °C and 90% relative humidity for 96 h. Subsequently, clean degreased gauze was employed to absorb the surface moisture from the specimens. Finally, the rate of length change for each specimen was calculated, and the test results were averaged across the six specimens.

The experiment utilized an ultra-depth-of-field three-dimensional microscope (VHX-6000) capable of accurately measuring 3D contours and volume, line roughness, and surface roughness, integrating observation and recording functions. Additionally, an ultra-depth-of-field optical microscope (KH-7700, Hirox Co., Ltd, Tokyo, Japan) was used to observe the macroscopic morphology of the GB and measure its surface roughness with a magnification range of 20–120 times. Surface roughness measurements were obtained from three surfaces of the sample (4 mm × 4 mm), focusing on parameters such as Sa, Sz, and Sq. Sa represents the arithmetic mean height of the scale-limited surface calculated as the arithmetic mean of the absolute ordinate values within a defined area. Sz represents the sum of the maximum peak and pit heights within a defined area.^34^ Another widely used and acknowledged surface parameter is Sq,^35^ which denotes the root mean square deviation of three-dimensional surface roughness. The reported results represent the average of measurements taken from at least three different positions on each specimen.

Static contact angle and surface free energy are vital parameters for coating applications. Contact angles were measured using two solvents (distilled water and diiodomethane) to examine the surface hydrophilicity and hydrophobicity at the temperature of the condensation reaction. Contact angle tests were conducted with a contact angle meter (Krüss, K11MK4, Germany) by dispensing 3 μL droplets onto the testing surface at room temperature. The results were averaged for at least six different positions on each specimen. Surface free energies of the dried wood coating were calculated using eqn (1) and (2):^36,37^1
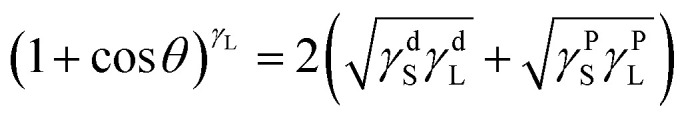

2 *γ* = *γ*^d^ + *γ*^P^

where *γ*_L_, *γ*^d^_L_, and *γ*^P^_L_ are the surface tension, dispersion force, and polarity force of the liquid, respectively; *γ*_S_, *γ*^d^_S_, and *γ*^P^_S_ are the surface tension, dispersion force, and polarity force of the solid,^16^ respectively; and *θ* is the contact angle of the dried coatings on the wood surface.

## 3 Result and discussion

### 3.1 Characterization of monomer and the resins

**Fig. 3:**
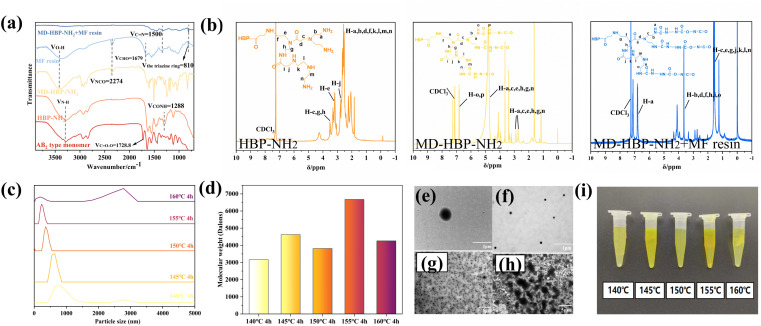
(a) Infrared spectra of MD-HBP-NH_2_ before and after synthesis and reaction. (b) ^1^H NMR of MD-HBP-NH_2_ before and after synthesis and reaction. (c) Particle size distribution of five MD-HBP-NH_2_s (nm). (d) The gel permeation chromatography (GPC) results of the prepared HBP-NH_2_s (Daltons). (e) and (f) Microscope photographs of TEMs of 155°C-HBP-NH_2_ (e) and 155°C-MD-HBP-NH_2_ (f) (the magnification was 15k and the scale was 1.0 μm). (g) and (h) Microscope photographs of TEMs of 140°C-HBP-NH_2_ (g) and 140°C-MD-HBP-NH_2_ (h) (the magnification was 2k and the scale was 5.0 μm). (i) Electronic photographs of the color of HBP-NH_2_ with the increase of polycondensation temperature.

To analyze and verify the occurrence and changes in the reaction system, the samples were characterized using FTIR (Fig. 3(a)). This characterization preliminarily confirmed the successful preparation of MD-HBP-NH_2_ using methyl acrylate (MA), diethylenetriamine (DETA), and the waterborne isocyanate SK-615. The peak at 1728.8 cm^−1^ in the monomer corresponded to the ester group O–C=O characteristic absorption peak, which completely disappeared in HBP-NH_2_, indicating the complete reaction of all ester bonds in MMA with DETA. Following the formation of HBP-NH_2_, a new peak emerged at 1288 cm^−1^, which was attributed to the absorption III band of –CONH–.^38,39^ Additionally, the characteristic absorption peak at 2274 cm^−1^ (ref. 40) represented the asymmetric stretching vibration of –NCO, confirming grafting of the isocyanate groups onto MD-HBP-NH_2_. These findings confirmed the successful synthesis of MD-HBP-NH_2_. In the MF resin, the characteristic absorption peak of the carbonyl group C=O in the CHO group was observed at 1679 cm^−1^.^41^ However, the absence of the –CHO group in the MD-HBP-NH_2_ blended MF resin suggested that it reacted with the amino group of MD-HBP-NH_2_. Furthermore, the presence of absorption peaks at 810 cm^−1^ (ref. 42) for the triazinyl ring and at 1500 cm^−1^ (ref. 43) for the carbon–nitrogen double bond in the MD-HBP-NH_2_/MF compound resin demonstrated the presence of the triazinyl ring and the reaction between amino groups in MD-HBP-NH_2_ and aldehyde groups in the melamine-formaldehyde resin.

Fig. 3(b) shows the ^1^H NMR spectra of HBP-NH_2_, MD-HBP-NH_2_, and MD-HBP-NH_2_ blended with MF resin. Compared with HBP-NH_2_, the spectrum of MD-HBP-NH_2_ exhibited significant changes. The four characteristic peaks at 2.7–2.9, 3.6, 7.8, and 3.4 ppm were assigned to (–CH_2_CH_2_–NH–CO–NH–R′, labeled b and m), (–CH_2_–NH–CO–NH–R′, labeled a and n), (–NH–CO–NH–R′, labeled o and p), and (–CH_2_CH_2_–NCO–CH_2_CH_2_)/(–CH_2_–NCO–CH_2_–), respectively. The presence of the peak at 4.8 ppm indicated (–NH–COO–) formation, suggesting the reaction of NCO functional groups with water. These results confirmed the successful synthesis of MD-HBP-NH_2_ with hyperbranched structures. After the reaction between MD-HBP-NH_2_ and the MF resin, the intensities of the four characteristic peaks of MD-HBP-NH_2_ notably diminished, suggesting a reaction between the –CHO of the MF resin and the-NH_2_ of MD-HBP-NH_2_. In this study, chloroform was used as the solvent for nuclear magnetic resonance (NMR) spectroscopy, owing to its excellent solubility in HBP-NH_2_. However, it is important to note that chloroform has limited solubility in MF resin, allowing only a portion of the soluble fraction to be extracted during NMR testing.

Fig. 3(c) presents the particle size statistics of MD-HBP-NH_2_. A notable trend emerged when the particle diameter of MD-HBP-NH_2_ increased with increasing temperature from 140 to 160 °C. Specifically, the particle diameter decreased from 700 to 400 nm with an increase in the condensation temperature from 140 to 145 °C. Subsequently, at the condensation temperature of 145 °C, the average particle diameter increased to 600 nm. Furthermore, upon reaching a condensation temperature of 155 °C, MD-HBP-NH_2_ assumed an appropriate structure with smaller particle sizes and optimal particle size distribution. As shown in Fig. 3(h), HBP-NH_2_ synthesized at a polymerization temperature of 140 °C exhibited both spherical and shuttle-shaped polymers. With an increase in polymerization temperature, HBP-NH_2_ obtained at 155 °C became more uniform and predominantly spherical, as illustrated in Fig. 3(g). The particle size distribution in Fig. 3(c) revealed that HBP-NH_2_ synthesized at 140 °C exhibited a bimodal distribution, likely due to increased agglomeration under these conditions. The particle size distribution in Fig. 3(c) showed that at 140 °C, HBP-NH_2_ displayed a shuttle shape, which increased the likelihood of agglomeration. In contrast, at temperatures between 145 °C and 155 °C, the distribution was more uniform with a single peak, indicating that higher polymerization temperatures promoted grafting and monomer conversion, resulting in a more uniform spherical polymer. The bimodal particle size distribution observed at 160 °C may be attributed to the increased surface energy and interfacial reactivity, which intensify interactions among hyperbranched polymers and promote agglomeration, thus leading to an apparent increase in particle size. In summary, the bimodal distributions at 140 °C and 160 °C suggest that the forces between these polymers hinder the homogeneity of droplet dispersion in wood modifier emulsion.

When the temperature of the condensation reaction exceeded 155 °C, the HBP-NH_2_ within the system reached its particle size limit. Consequently, there was no further decrease in the particle size. The particle size exhibited two peaks with wide distribution, averaging at 200 nm and 2750 nm. Despite this, the polydispersity remained unchanged, indicating complete reaction saturation of the intermediates. Therefore, exceeding a certain temperature threshold during condensation can affect the particle size and distribution. The observed non-uniformity in the particle diameters of 160°C-HBP-NH_2_ may be attributed to agglomeration during polymerization.

The average molecular weights of the various HBP-NH_2_ samples, as determined by GPC and presented in Fig. 3(d) and Table 2, revealed distinct trends. Based on previous studies,^44^ polymer degradation is typically initiated by chain fragmentation, followed by branching, cross-linking, and the formation of double bonds. As depicted in Fig. 3(d), the *M*_n_ value of HBP-NH_2_ increased from 140 °C to 145 °C owing to the maturation cross-linking of residual functional groups within the molecule under higher temperature polycondensation conditions.^45^ In contrast, the *M*_n_ value decreased for the 150°C-HBP-NH_2_ sample, suggesting chemical bond rupture and a reduction in molecular weight, whereas *M*_n_ increased for the 155°C-HBP-NH_2_ sample, indicating intermolecular polymerization. For the 160°C-HBP-NH_2_ sample, the polydispersity remained nearly constant, suggesting near completion of the polycondensation reaction, whereas *M*_n_ decreased. Compared with the 155°C-HBP-NH_2_ sample, the molecular weight of the 160°C-HBP-NH_2_ sample decreased to 4262 Da. This reduction could be attributed to the formation of a regular three-dimensional structure resembling a nearly spherical shape as the molecular weight of HBP-NH_2_ reached a certain degree. Owing to its non-twisted configuration, this structure offers less fluid resistance than that of ordinary linear polymers. The decrease in the molecular weight, as measured by GPC, was likely due to the smaller volume of the molecules being measured.

In addition, the size variation of the wood modifier MD-HBP-NH_2_ was observed using TEM. The TEM images in Fig. 3(e) and (g) illustrate that the prepared HBP-NH_2_ exhibited a smooth surface and a spherical shape with a more uniform particle size distribution.

To examine the microstructure of the hyperbranched polymers, TEM tests were conducted on 155°C-HBP-NH_2_ and 155°C-MD-HBP-NH_2_. The results are depicted in Fig. 3(e) and (f), revealing a spherical shape in the solution micro-morphology. The particle sizes of 155°C-HBP-NH_2_ and 155°C-HBP-NH_2_ were concentrated at 200–400 nm and 500–1500 nm, respectively. This is consistent with the results of the particle-size test. TEM analysis indicated that the addition of the aqueous isocyanate cross-linker SK-615 did not alter the morphology of HBP-NH_2_ but affected the particle size and distribution. The TEM patterns revealed that the addition of SK-615 led to a gradual decrease in the solution particle size and reduced agglomeration, indicating effective dispersion of HBP-NH_2_. However, excessive polycondensation results in increased agglomeration and a larger particle size. TEM results further demonstrated that the dispersion of SK-615 in 140°C-HBP-NH_2_ was suboptimal, with attachment and agglomeration phenomena observed.

Fig. 3(g) and (h) show the TEM results, indicating that HBP-NH_2_ and MD-HBP-NH_2_ formed at 140 °C reached micrometer-scale lengths. Specifically, 140°C-MD-HBP-NH_2_ exhibited primarily rod and globular shapes. The transition from a rod to a globular shape in 140°C-HBP-NH_2_ was attributed to the low degree of polymerization of 140°C-HBP-NH_2_, where an increase in the branching degree led to structural changes and a gradual reduction in molecular size.

As the degree of branching increased, the microstructure of MD-HBP-NH_2_ changed from rod-like to spherical, and the molecular size gradually decreased. Among the five emulsions prepared, those formed using 155°C-MD-HBP-NH_2_ exhibited smaller emulsion particle sizes and superior particle size distributions. The molecular chains with minimal entanglement exerted little influence on the emulsion particle size, resulting in fluctuations within a certain range. Concurrently, the TEM images (Fig. 3(e)–(h)) confirmed the observed distribution pattern of the particle size and enhanced compatibility of SK-615 with HBP-NH_2_.

The particle diameters of MD-HBP-NH_2_ at temperatures ranging from 140 °C to 160 °C are illustrated in Fig. 3(c), demonstrating the relationship between the particle diameters of MD-HBP-NH_2_ and the temperature of the condensation reaction. Notably, both 140°C-MD-HBP-NH_2_ and 160°C-MD-HBP-NH_2_ exhibited significant agglomeration, which might adversely affect the resin formation.

The figure depicts HBP-NH_2_ synthesized at various polycondensation temperatures, and Table 1 presents the average particle sizes (P10, P50, and P100) of MD-HBP-NH_2_ as a function of increasing polycondensation temperature. The samples displayed distinct color variations with noticeable differences in the uniformity and transparency of their color distributions.

**Table 1:** Average particle size, P10, P50, P100 of MD-HBP-NH_2_s with the increase of polycondensation temperature. *E.g.*: the particle size of 10% of 140°C-MD-HBP-NH_2_ is less than 553.2

Polymer	Average particle size	P10	P50	P100
140°C-MD-HBP-NH_2_	851.0	553.2	741.9	2780
145°C-MD-HBP-NH_2_	605.2	477.7	640.7	741.9
150°C-MD-HBP-NH_2_	346.1	265.6	356.2	477.7
155°C-MD-HBP-NH_2_	238.6	171	229.3	356.2
160°C-MD-HBP-NH_2_	910.4	52.85	198	2780

**Table 2:** Molecular weight and polydispersity index (PDI) of HBP-NH_2_s with the increase of polycondensation temperature

Polymer	*M*_n_ (daltons)	Polydispersity
SK615	1546	2.033172
140°C-HBP-NH_2_	3165	2.263581
145°C-HBP-NH_2_	4623	1.966104
150°C-HBP-NH_2_	3813	1.750199
150°C-HBP-NH_2_	6676	1.854023
160°C-HBP-NH_2_	4262	2.169961

### 3.2 Surface morphology and physical performance of the modified wood surface

The experiment employed an ultra-depth-of-field three-dimensional microscope (VHX-6000) capable of accurately measuring 3D contours and volume, line roughness, and surface roughness, thereby integrating observation and recording.

**Fig. 4:**
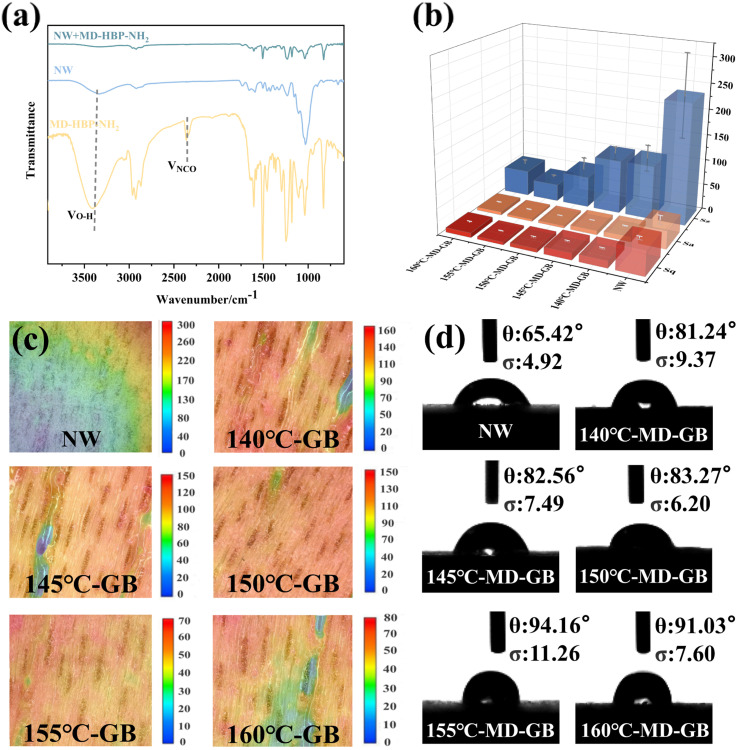
(a) Infrared spectra of MD-HBP-NH_2_ after reaction. (b) Surface roughness measurement results (Sa, Sz and Sq). (c) Surface 3D topography of the samples. (d) The initial water contact angle and the water contact angle after five seconds.

In Fig. 4(a), the FT-IR spectrum illustrates the interface between the natural wood (NW) and grafted board (GB). The peak at 3300 cm^−1^ corresponded to the stretching vibration of –OH in natural wood. In GB, the hydroxyl group peak at 3330 cm^−1^ and the –NCO peak at 2274 cm^−1^ nearly disappeared, indicating a substantial reaction between the hydroxyl and isocyanate groups of MD-HBP-NH_2_.

Fig. 4(b) presents the primary roughness measurements (Sa, Sz, and Sq) of the GB surface at various condensation temperatures. These results indicated that as the condensation temperature increased from 140°C-MD-HBP-NH_2_-GB to 155°C-MD-HBP-NH_2_-GB, the surface roughness decreased. The application of various polymers produced polymer film surfaces with varying degrees of flatness, which improved the surface roughness of wood to varying extents. However, with the transition from 155°C-MD-HBP-NH_2_-GB to 160°C-MD-HBP-NH_2_-GB, only some polymers effectively filled the voids in wood. The remainder formed agglomerates on the wood surface, leading to a coarser structure and increased roughness of the grafted board. These findings indicated that as the particle size decreased, the surface roughness of the GB decreased, implying that reducing the particle size of HBP-NH_2_ may help minimize the surface roughness of the GB and enhance its morphology. The optimal surface morphology and the lowest surface roughness were achieved at a condensation temperature of 155 °C.

Because of the ridge-like raised cell walls and groove-like cell cavities on the wood surface along with surface cracks caused by unbalanced growth stress during timber processing, the veneer exhibited low flatness. Fig. 4(c) illustrates 3D micrographs of wood surfaces coated with various hyperbranched polymers. Different colors denote distinct depths of field in the model, with uniform coloration indicating surface flatness. The surface of the NW was rough, as evident from the color map displaying numerous pronounced grooves in the surface profile height.

140°C-MD-HBP-NH_2_-GB and 145°C-MD-HBP-NH_2_-GB exhibited a reddish surface color, with blue and green hues visible in the color map of the surface profile height. Different colors in the 3D model, representing varying depths of field, could indicate a considerable difference in the surface flatness uniformity. Lighter-colored grooves indicated incomplete filling. MD-HBP-NH_2_ formed a noticeable surface layer on wood, predominantly displaying a red hue. In comparison, the surfaces of 150 and 155°C-MD-HBP-NH_2_-GB were flatter and more uniformly colored. Different colors in the 3D model reflected the different depths of the field, highlighting the film's flatness and uniformity. The reduction in Sz compared with NW was evident, with reductions of 50% and 76.67%, respectively. The deepening color of the surface grooves primarily resulted from the smaller particle size of MD-HBP-NH_2_ after completion, which ensured a more uniform distribution within the grooves. As a result, most conduits were filled, which significantly reduced surface roughness. However, from 155°C-MD-HBP-NH_2_-GB to 160°C-MD-HBP-NH_2_-GB, only a portion of the conduits was filled, while the remainder formed agglomerates on the wood surface, resulting in incomplete filling. The comparison of the super-depth-of-field 3D maps in Fig. 4(c) indicated the following order of color: 155°C-MD-HBP-NH_2_-GB > 150°C-MD-HBP-NH_2_-GB > 160°C-MD-HBP-NH_2_-GB > 145°C-MD-HBP-NH_2_-GB > 140°C-MD-HBP-NH_2_-GB > NW.

The contact angle between distilled water and the wood surface was used to evaluate the hydrophobicity of the modified wood. To precisely depict the change in contact angle, images of the contact angle between distilled water and wood were captured and analyzed individually for the first second.

Fig. 4(d) displays the initial water contact angle results for all the GBs. The initial contact angle of the NW was the lowest. According to the chemical composition, wood consists primarily of lignin, cellulose, and hemicellulose.^46^ Cellulose and hemicellulose are notably rich in hydroxyl groups, rendering them highly hydrophilic. From the experiment, the initial contact angle between the distilled water and NW was measured to be 65.42° within the first second.

When combined with 140°C-MD-HBP-NH_2_-GB, 145°C-MD-HBP-NH_2_-GB, and 150°C-MD-HBP-NH_2_-GB with NW, distilled water exhibited lower wetting on the modified wood, resulting in a larger contact angle within the first second. This difference in contact angle between the grafted and native wood can be attributed to the coating chemistry, which is much more hydrophobic than the wood itself. Consequently, the modified wood exhibited enhanced hydrophobicity, leading to an increase in the water contact angle.

Notably, the initial contact angles of 155°C-MD-HBP-NH_2_-GB and 160°C-MD-HBP-NH_2_-GB exceeded 90°, indicating a shift towards hydrophobic behavior compared to the hydrophilic state. This suggested that the 155°C-MD-HBP-NH_2_-GB coating exhibited reduced susceptibility to water dissolution and penetration. As the polycondensation temperature increased, the contact angles within the first second also increased, demonstrating enhanced resistance to water swelling and penetration for 155°C-MD-HBP-NH_2_-GB and 160°C-MD-HBP-NH_2_-GB. This was attributed to the formation of a cross-linked mesh structure by the hyperbranched polymers, which mitigated the swelling effect of water.^47^ Specifically, the contact angle of distilled water with 155-GB was 94.16° within the first second.

In summary, MD-HBP-NH_2_ enhanced the hydrophobicity of wood. The high hydrophobicity of 155°C-MD-HBP-NH_2_-GB was due to the surface grafting of isocyanate, which effectively sealed off surface hydroxyl groups. Similar results were observed for 160°C-MD-HBP-NH_2_-GB. Conversely, 140°C-MD-HBP-NH_2_-GB, 145°C-MD-HBP-NH_2_-GB, and 150°C-MD-HBP-NH_2_-GB exhibited smaller contact angles, likely because MD-HBP-NH_2_ formed at lower temperatures, with better water solubility and a lower degree of cross-linking than that formed at 155 °C.^48^

The results of the water and diiodomethane static contact angle tests for NW, impregnated decorative paper, and GBs are presented in Table 3, along with their corresponding surface free energies shown in Table 4. The initial contact angle was measured at 0. As the condensation temperature increased, the contact angles of the materials increased, whereas the surface free energy decreased. This trend can be attributed to the impact of the increased molecular weight and branching degree on the polymer chain size and mobility.^19^ The wettability of the material decreased significantly as the molecular size increased. However, at a condensation temperature of 160 °C, the contact angle decreased, and the surface free energy increased. This shift may result from increased molecular chain rigidity and reduced chain entanglement at excessively high condensation temperatures.^49^ When the surface free energies of the two substances closely matched, they were more likely to blend. Among the five GBs, the surface free energy of 140°C-MD-HBP-NH_2_-GB was the highest, whereas that of 155°C-MD-HBP-NH_2_-GB was the lowest. The surface free energy of the hot-pressed impregnated decorative paper was the lowest, indicating good wettability between the 155°C-MD-HBP-NH_2_-GB coating and hot-pressed impregnated paper. This suggested that the wood coating effectively adhered to the impregnated decorative paper, thereby enhancing the overall bonding force between the two materials.

**Table 3:** Initial contact angles of different reference liquids on the surface of natural wood (NW) and MD-HBP-NH_2_ grafted board (GB)

Liquid label	NW	Impregnated decorative paper	Impregnated decorative paper after hot pressing	140°C-MD-HBP-NH_2_-GB	145°C-MD-HBP-NH_2_-GB	150°C-MD-HBP-NH_2_-GB	155°C-MD-HBP-NH_2_-GB	160°C-MD-HBP-NH_2_-GB
Distilled water (°)	65.42	64.35	95.42	81.24	82.56	83.27	94.16	91.03
Diiodomethane (°)	57.75	53.78	55.93	42.88	43.60	48.42	52.77	43.81

**Table 4:** Dispersion (*γ*^d^_S_) and polar (*γ*^P^_S_) surface tension components of different reference liquids on the surface of natural wood (NW) and MD-HBP-NH_2_ grafted board (GB)^a^

*γ* (mJ m^−2^) label	NW	Impregnated decorative paper	Impregnated decorative paper after hot pressing	140°C-MD-HBP-NH_2_-GB	145°C-MD-HBP-NH_2_-GB	150°C-MD-HBP-NH_2_-GB	155°C-MD-HBP-NH_2_-GB	160°C-MD-HBP-NH_2_-GB
*γ* ^d^_S_	29.87	32.14	30.91	38.13	37.75	35.15	32.72	37.64
*γ* ^P^_S_	13.28	12.94	0.97	3.37	3.03	3.31	0.98	0.99
*γ*_S_	43.15	45.08	31.88	41.5	40.78	38.46	33.70	38.63

a P.S.: the reference liquids used were distilled water and diiodomethane.

Wood, a natural hygroscopic material, is prone to deformation owing to its inherent properties of dry shrinkage and wet swelling during usage.^50^

At a high temperature (70 °C), the chordwise dimensional change rate of the NW was approximately 0.266%, whereas at high humidity (40 °C, 90% RH), it was approximately 0.198%. The total dimensional change rate was 0.464%.

Table 5 illustrates the impact of various hyperbranched polymers grafted onto wood on the rates of dimensional change under high heat and humidity conditions.

**Table 5:** Dimensional stability of GB

Testing method label	NW	140°C-MD-HBP-NH_2_-GB	145°C-MD-HBP-NH_2_-GB	150°C-MD-HBP-NH_2_-GB	155°C-MD-HBP-NH_2_-GB	160°C-MD-HBP-NH_2_-GB
Dry heat test (%)	0.266	0.221	0.178	0.217	0.127	0.131
High humidity test (%)	0.198	0.120	0.160	0.078	0.227	0.21
Sum (%)	0.464	0.341	0.338	0.296	0.354	0.341

Table 5 shows that the dimensional change rates of the five GBs varied under high heat (70 °C) and high humidity (40 °C, 90% RH) conditions compared with NW. As the condensation temperature increased, the rate of dimensional change under high-heat conditions initially decreased from 0.221% to 0.178%, then fluctuated to 0.217%, followed by a decrease to 0.127%, and finally increased to 0.131%. Nevertheless, the trend in the change under high-humidity conditions was contrary to that observed under high-heat conditions.

During the heating process, the wood underwent thermal action, causing water evaporation and simultaneous chemical compositional changes in the wood cell wall. This involves a reduction in hydrophilic groups, such as hydroxyl groups,^51,52^ leading to the generation of numerous free radicals and the generation of new hydrophobic constituents. Consequently, these chemical alterations resulted in wood shrinkage due to water loss post-heating, significantly affecting its physical properties under high heat conditions, as evidenced by mass loss and dimensional shrinkage (manifested as negative rates of dimensional change in the chordal direction).

Under high humidity conditions, the free hydrophilic groups in wood bind numerous water molecules, causing cellulose molecular chains to space out more widely. This resulted in macroscopic dimensional expansion of the wood, which was evident as a positive rate of dimensional change in the chordal direction.

The hydroxyl groups within the wood interact with the isocyanate groups present in hyperbranched polymers. The hydrophilic hydroxyl groups in wood were replaced with hydrophobic carbamate bonds, consequently reducing the moisture absorption capacity of the wood.

Furthermore, between 150°C-MD-HBP-NH_2_ and 155°C-MD-HBP-NH_2_, the wood surface exhibited the most complete reactions, with the fewest free hygroscopic groups and fewer hydrophilic groups reduced by bonding with water molecules. Consequently, these variants demonstrated the lowest rates of dimensional change under high-humidity and high-heat conditions.

After grafting onto MD-HBP-NH_2_, the high-temperature dimensional change rate of 155°C-MD-HBP-NH_2_-GB decreased by 52.26%, whereas the high-humidity dimensional change rate decreased by 60.61%. Additionally, the total dimensional change rate of 150°C-MD-HBP-NH_2_-GB decreased by 36.21%.

### 3.3 Surface properties of the impregnated decorative paper bonded panels

The surface bonding strength indicated the quality of adhesion between the surface decorative layer and the impregnated decorative paper-laminated wood substrate. Poor bonding quality increased the risk of peeling the decorative layers.

**Fig. 5:**
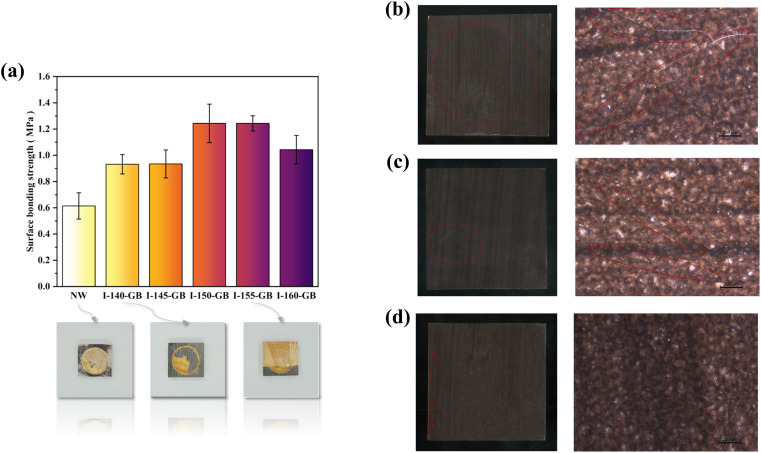
(a) Surface bonding strength of wood bonded with impregnated decorative paper and digital pictures of damaged samples after surface bonding test. Digital and microscopic pictures (the magnification was 4 and the scale was 200 μm) of I-NW samples after surface cold and hot cycling resistance test. (b) I-NW. (c) I-145°C-MD-HBP-NH_2_-GB. (d) I-160°C-MD-HBP-NH_2_-GB.

Fig. 5(a) illustrates the surface bond strengths of various hyperbranched polymer-grafted blockboards impregnated with decorative paper. According to GB/T34722-2017 standards for “Surface decorated plywood and blockboard with paper impregnated thermosetting resins”, the surface bond strength of impregnated decorative paper blockboards should be equal to or greater than 0.6 MPa.

The bond strength between the impregnated decorative paper and NW was merely 0.61 MPa, as shown in Fig. 5, depicting digital image post-tests. As illustrated in Fig. 5(a), the control group exhibited a low bond strength between the impregnated decorative paper and NW, with specimen damage occurring primarily at the interface. Notably, the MF resin at the fracture interface was visibly disrupted.

Fig. 5(a) shows the interface damage observed after the bonding strength test between the impregnated decorative paper and 140-GB. The irregularity of the damaged interface was notable, with some wood fibers evident on the impregnated decorative paper after the test. This suggested that the hyperbranched polymer not only bonded to the wood surface, but also possessed a bond strength exceeding that of the polymer and MF resin itself, indicative of a robust cross-linking structure.

After grafting various MD-HBP-NH_2_ onto the wood surface, the surface bonding strength of the I-GB increased to varying degrees. Moreover, with an increase in the condensation temperature of the hyperbranched polymer, there was a more pronounced enhancement in the bonding strength.

As the condensation temperature of the hyperbranched polymer increased, the enhancement in bonding strength became more pronounced.

During the grafting process of 150°C-MD-HBP-NH_2_/155°C-MD-HBP-NH_2_ onto the wood surface, wood fibers were notably present on the impregnated decorative paper after the test, as depicted in Fig. 5(a). The specimen damage from the surface bonding strength test primarily affected the veneer of the blockboard, with minimal damage to the bond layer. The surface bonding strength of I-GB reached 1.24 MPa, significantly surpassing national standard requirements. This enhancement can be attributed to the introduction of MD-HBP-NH_2_, which introduced active groups such as isocyanate and amino groups in the intermediate interface layer. These groups facilitated reactions with the hydroxyl groups on the wood veneer and MF resin in the impregnated decorative paper, forming a crosslinked structure that substantially improved the bonding between the wood veneer and impregnated decorative paper.

Without MD-HBP-NH_2_, the bonding between the impregnated decorative paper and veneer in the surface layer relied only on the MF resin, resulting in a naturally low surface bonding strength. However, grafting with 150 °C and 155°C-MD-HBP-NH_2_ significantly enhanced the adhesive strength. Conversely, grafting with 140°C-MD-HBP-NH_2_ led to a decrease in surface adhesive strength. This decline may stem from the lower molecular weight and inadequate reaction of the wood hydroxyl groups owing to the condensation conditions at 140 °C for 4 h, weakening the combination of the wood veneer and impregnated paper. Conversely, increasing the polymerization temperature to 155 °C resulted in maximum surface bonding strength in this group of specimens.

Compared with the aforementioned cases, the inclusion of MD-HBP-NH_2_ at 160 °C for 4 h also enhanced the adhesive strength. However, an excessively high condensation temperature led to unfavorable conditions for the reaction with the wood hydroxyl groups.

The tests evaluated the surface cracking resistance and resistance of the I-GB to cold and hot cycling, and the results are presented in Table 6. The joinery substrate consists of small wood strips and rotary-cut veneers, which are known for their dimensional instability. During testing for cold and heat cycle resistance and cracking, significant moisture content variations were observed in the specimens. However, the dry shrinkage or wet rise of the impregnated decorative paper did not align with that of the substrate, leading to surface cracking. To examine the crack distribution and patterns after the hot and cold cycling tests, the I-GB surface was analyzed through visual observation and optical microscopy (Leica S6D), as shown in Fig. 5(b)–(d). The analysis revealed obvious cracks in both I-NW and I-145°C-MD-HBP-NH2-GB specimens, with the cracks in I-NW penetrating through the sample, whereas I-160°C-MD-HBP-NH_2_-GB showed almost no cracks. Additionally, the results of the cracking resistance test and the hot and cold cycling tests demonstrated a substantial improvement when the wood surface was treated with HBP-NH_2_ and SK615 dispersions compared to treatment with SK615 alone. Specifically, specimens treated only with SK615 exhibited 7 and 6 cracks, respectively, whereas those treated with dispersion showed a significant reduction in crack incidence. These findings highlight that the enhanced crack resistance of the I-GB formulation is not solely attributable to the hyperbranched polymer or SK615 individually but rather stems from the synergistic interaction between HBP-NH_2_ and SK615 within the dispersion.

**Table 6:** Effect of modifiers on surface cracking resistance and surface cold and hot cycling resistance of I-GB

Test (number of crack) specimen	I-NW	I-SK615-GB	I-140°C-MD-HBP-NH_2_-GB	I-145°C-MD-HBP-NH_2_-GB	I-150°C-MD-HBP-NH_2_-GB	I-155°C-MD-HBP-NH_2_-GB	I-160°C-MD-HBP-NH_2_-GB
Cracking resistance	29	7	11	10	6	2	1
Cold and hot cycling resistance	13	6	4	5	0	0	3

Table 6 shows a gradual reduction in the number of cracks with increasing condensation temperature of the hyperbranched polymer. This indicated that the addition of MD-HBP-NH_2_ effectively enhanced the cracking resistance of I-GB, leading to a decrease in the number of cracks, which was consistent with its resistance to cold and hot cycles. Notably, when the polycondensation temperature was increased to 150 °C, the number of cracks decreased sharply to almost zero. According to GB/T 17657-2022, surface cracks resulting from edge damage are not considered when evaluating surface cracking conditions.

### 3.4 Observation of the mechanism of action and microstructure

SEM was used to examine the wood surface and interface before and after grafting and hot-pressing. The notable differences in dimensional stability, surface free energy, and surface roughness between NW (natural wood) and 155°C-MD-HBP-NH_2_-GB contributed to the different results of the surface property tests of the two types of surface-decorated boards.

SEM was employed to examine the wood surface and interface before and after grafting and hot pressing. The significant differences in dimensional stability, surface free energy, and surface roughness between the NW and 155°C-MD-HBP-NH_2_-GB resulted in different outcomes in the surface property tests of the two types of surface-decorated boards.

**Fig. 6:**
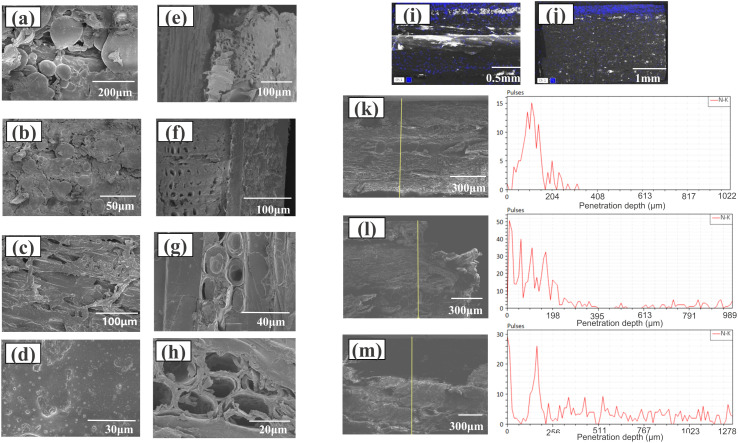
Surface and interface morphology of the blockboard after gluing and hot pressing (a). Surface of nature wood (NW)(700×). (b) Surface of nature wood (NW)(2000×). (c) Surface of 155°C-MD-HBP-NH_2_-GB(400×). (d) Surface of 155°C-MD-HBP-NH_2_-GB(1800×). (e) Bonding interface between impregnated decorative paper and NW(220×). (f) Bonding interface between impregnated decorative paper and 155°C-MD-HBP-NH_2_-GB(400×). (g) Morphology of wood cell structure at the binding interface region between 155°C-MD-HBP-NH_2_-GB and impregnated decorative paper (1300×). (h) Morphology of wood cell structure in the bonding interface region between NW and impregnated decorative paper (2000×). (i) SEM-EDS area scan map of N element along the depth of I-NW. (j) SEM-EDS area scan map of N element along the depth of I-155°C-HBP-NH_2_-GB(one layer). (k) SEM-EDS line scan map of N element along the depth of I-NW. (l) SEM-EDS line scan map of N element along the depth of I-140°C-MD-HBP-NH_2_-GB. (m) SEM-EDS line scan map of N element along the depth of I-155°C-MD-HBP-NH_2_-GB.

Fig. 6(a)–(d) show the variations in the topography and morphology of the ungrafted and grafted Eucalyptus surfaces. This comparison demonstrated the morphological changes between NW and 155°C-MD-HBP-NH_2_-GB. Specifically, in Fig. 6(a) and (b), the surface was rough and uneven, characterized by ridge-like raised cell walls, grooved cell cavities, and microcracks resulting from the initial drying of the wood.

In the high- and low-magnification images in Fig. 6(c) and (d), the microstructure of 155°C-MD-HBP-NH_2_/GB was largely concealed beneath a smooth and uniform coating that extended across the entire surface without cracks or micropores.

The SEM images of I-NW and 155°C-MD-HBP-NH_2_-GB are presented in Fig. 6(e) and (f). In the sample without adhesive, a gap of approximately 20 μm was observed between the impregnated decorative paper and substrate. Conversely, the substrate treated with 155°C-MD-HBP-NH_2_ exhibited seamless connection with the impregnated decorative paper. These findings align with those of the surface bonding strength test, confirming that MD-HBP-NH_2_ facilitated the formation of an efficient connection layer between the impregnated decorative paper with MF resin and wood surface, thereby enhancing their interfacial bonding.

Previous experimental results^53,54^ have demonstrated that, during the process, bulking chemicals diffuse into the cell walls and augment their volume, particularly on wood surfaces grafted with MD-HBP-NH_2_.

SEM was employed to investigate the impact of MD-HBP-NH_2_ on the microstructure of wood and the distribution of modifiers (Fig. 6(g)). The cross section of a natural wood cell wall is shown in Fig. 6(h). As shown in Fig. 6(g), 155°C-MD-HBP-NH_2_ covered the cell lumen and the gap between cells. Compared to untreated wood (Fig. 6(h)), the original honeycomb cellular structure remained intact, whereas the cell wall thickness increased because of the wetting and swelling effects of MD-HBP-NH_2_ on the cell wall. The infiltration of MD-HBP-NH_2_ into the wood cell wall improved its hydrophobicity and dimensional stability by wetting the cell wall and reacting with the hydroxyl groups of the wood.

Compared to NW, the morphology of the cell wall structure in the chemically bonded interface region of the GB underwent significant changes. The wood cell wall transitioned from a porous to a smooth and dense structure, indicating that the adhesive penetrated the wood cell wall and formed bonds at the nanoscale.

Grafting wood with MD-HBP-NH_2_ significantly enhanced the surface bonding strength of melamine-formaldehyde-impregnated decorative paper and wood to 1.24 MPa. To explore the microstructure of the N elements within the hyperbranched polymer at various depths from the wood surface under these conditions, EDS area and line scanning analyses were conducted on the I-NW and I-GBs. The distribution results for N are shown in Fig. 6(i)–(m).

In Fig. 6(i), the morphology and distribution of N at the interface of the I-NW-impregnated decorative paper/NW are depicted. The analysis revealed that the majority of N elements were concentrated at a depth of approximately 0.2 mm, corresponding to the thickness of the impregnated decorative paper. N elements observed below 0.2 mm likely originated from penetration into the wood. At this interface, the boundary between the impregnated decorative paper and NW appeared indistinct, with no clear reaction layer.

In Fig. 6(j), the boundary between N and NW was clearer in the case of I-155°C-MD-HBP-NH_2_-GB than the thickness of the impregnated decorative paper. This clarity resulted from the increased N content of the wood after finishing. Fig. 6(k) illustrates the line scan of the I-NW, revealing that most of the N was concentrated within a thickness of 300 μm (slightly larger than the impregnated decorative paper), with relatively lower concentrations observed below 300 μm depth, mainly from the penetration of the triamine adhesive. Fig. 6(l) and (m) depict the results of the interfacial line scanning of the I-140°C-MD-HBP-NH_2_-GB and I-155°C-MD-HBP-NH_2_-GB joints, respectively. Owing to the coating of the hyperbranched polymer, the N element peak on the wood surface increased, and the thickness of the N element penetration increased. However, the increase was not significant for I-140°C-MD-HBP-NH_2_-GB, which had a penetration thickness of 400 μm. Conversely, for 155°C-MD-HBP-NH_2_-GB, the N element peak on the wood surface decreased, whereas the thickness of the N element penetration significantly increased. Additionally, the penetration layer thickness increased notably with grafting of the hyperbranched polymer. In the case of 155°C-MD-HBP-NH_2_-GB, the N peak on the wood surface decreased, and the N penetration thickness increased significantly, gradually transitioning to a uniform distribution with depth. This uniform distribution and the decrease in the signal depth in the line scans indicated the successful grafting of MD-HBP-NH_2_ and the gradient change of N within the wood interior.

## 4 Conclusion

In conclusion, this study reported a waterborne hyperbranched polyurethane that offered outstanding crack resistance to surface-decorated blockboards. The straightforward grafting method, which involved applying a direct finish to the joinery surface to impart a low surface energy similar to that of impregnated decorative paper, resulted in excellent dimensional stability and hydrophobicity of the wood. This method improved the surface quality of the wood, promoted bonding between the blockboard and the impregnated decorative paper, and effectively prevented cracking. The modifier penetrated the wood cell wall and cavity, establishing hydrogen bonding between the isocyanate group of the main chain and numerous exposed hydroxyl groups, thereby forming enduring adhesion to the wood surface. The construction of a hyperbranched “root” structure, along with the branching of terminal amino acids into the impregnated decorative paper (“soil”) during hot pressing, further enhanced the surface bonding strength. Optimal comprehensive performance was achieved at a polycondensation reaction temperature of 155 °C for 4 h. Compared with the untreated group, the water contact angle increased from 68.01° to 94.16°, rendering the surface flatter. Moreover, the high thermal and humidity stabilities were notably improved, with reductions of 52.26% and 60.61%, respectively, compared with the control group. Additionally, the surface-decorated blockboards modified with paper-impregnated MF resin exhibited significantly enhanced surface bond strength, cracking resistance, and resistance to hot and cold cycles.

## Data availability

The data underlying this article are available in this article and in its ESI.†

## Conflicts of interest

The authors declare no conflicts of interest.

## Supplementary Material

RA-014-D4RA07688E-s001

RA-014-D4RA07688E-s002
