# Supplementary material for: Root-inspired grafting of wood surfaces with hyperbranched polymers for enhanced interfacial adhesion with impregnated decorative paper
Source: RSC Adv. 2024 Dec 9;14(52):38706–20. doi: 10.1039/d4ra07688e (PMC11626439; doi:10.1039/d4ra07688e)
Supplement: RA-014-D4RA07688E-s001 [file RA-014-D4RA07688E-s001.pdf]

Supplementary Information (SI) for RSC Advances.
This journal is © The Royal Society of Chemistry 2024

Root-inspired grafting of wood surfaces with hyperbranched polymers for enhanced interfacial adhesion with impregnated decorative paper

Hui Li[a, b], Ru Liu,[b,*] Xinyu Li[c] , Kun Xu[c], Jinzhen Cao, [a,*]

a.School of Materials Science and Technology, Beijing Key Laboratory of Wooden Material Science and Application, Beijing Forestry University, Haidian, Beijing 100083, China.

Email: caoj@bjfu.edu.cn

b.Institute of Wood Industry, China Academy of Forestry Sciences, Haidian, Beijing 100091, China.

E-mail: liuru@criwi.org.cn

c.Guangdong Tianyuan Huibang New Materials Co., Ltd, Foshan, Guangdong 528100, China.

Keyword: Wood surface; Root-structural hyperbranched polymer; Impregnated film paper; Interfacial adhesion; Crack-resistance

It is noteworthy that this video characterization method was used in the experimental work on cracking resistance test. Firstly, it was used to set the temperature of the blast drying oven to 70°C. After that, I-160°C-HBP-NH_2-GB and I-NW were passed into the oven for 24 hours, respectively, and finally, they were taken out of the oven and the number of cracks was counted. From the video, it can be observed that I-160°C-HBP-NH_2-GB did not produce cracks, while I-NW produced a number of cracks. This means that a waterborne hyperbranched polyurethane that offered outstanding crack resistance to surface-decorated blockboards was made.
